# Which Effects on Neuroanatomy and Path-Integration Survive? Results of a Randomized Controlled Study on Intensive Balance Training

**DOI:** 10.3390/brainsci10040210

**Published:** 2020-04-03

**Authors:** Milos Dordevic, Marco Taubert, Patrick Müller, Martin Riemer, Jörn Kaufmann, Anita Hökelmann, Notger G. Müller

**Affiliations:** 1 German Center for Neurodegenerative Diseases (DZNE), 39120 Magdeburg, Germany; Patrick.Mueller@dzne.de (P.M.); martin.riemer@dzne.de (M.R.); notger.mueller@dzne.de (N.G.M.); 2 Center for Behavioral Brain Sciences (CBBS), 39106 Magdeburg, Germany; marco.taubert@ovgu.de; 3 Neurology Clinic, Otto von Guericke University, 39120 Magdeburg, Germany; joern.kaufmann@med.ovgu.de; 4 Chair for Training Science “Cognition and Motion”, Department Sports Science, Otto von Guericke University, 39104 Magdeburg, Germany; anita.hoekelmann@ovgu.de

**Keywords:** neuroplasticity, path integration, VBM, gray matter, balance

## Abstract

Balancing is a complex task requiring the integration of visual, somatosensory and vestibular inputs. The vestibular system is linked to the hippocampus, a brain structure crucial for spatial orientation. Here we tested the immediate and sustained effects of a one-month-long slackline training program on balancing and orientation abilities as well as on brain volumes in young adults without any prior experience in that skill. On the corrected level, we could not find any interaction effects for brain volumes, but the effect sizes were small to medium. A subsequent within-training-group analysis revealed volumetric increments within the somatosensory cortex and decrements within posterior insula, cerebellum and putamen remained stable over time. No significant interaction effects were observed on the clinical balance and the spatial orientation task two months after the training period (follow-up). We interpret these findings as a shift away from processes crucial for automatized motor output towards processes related to voluntarily controlled movements. The decrease in insular volume in the training group we propose to result from multisensory interaction of the vestibular with the visual and somatosensory systems. The discrepancy between sustained effects in the brain of the training group on the one hand and transient benefits in function on the other may indicate that for the latter to be retained a longer-term practice is required.

## 1. Introduction

It is known that many new movements, especially when learned for the first time, can cause both transient and sustained structural brain changes, which can often be detected using voxel-based morphometry (VBM) [1,2,3,4,5,6]. The ability of the human nervous system to adapt to new experiences by altering its connectivity and creating new neurons (in areas such as the hippocampus), a process termed neuroplasticity, has been confirmed by numerous previous studies that used movement interventions as stimulus [7,8]. There is still an ongoing debate on the localization of the “vestibular cortex”, as this modality does not have a specific sensory area within the brain, making it plausible for several brain regions to be affected by a training that strongly stimulates the vestibular system, such as slackline training (balancing on a narrow ribbon). Slackline training does not differ much from other balance trainings in terms of the involved neuroanatomical structures; however, it is more intensive and should, therefore, lead to faster and stronger effects.

Identifiable downsides of previous studies on this matter include the cross-sectional nature of research and the inhomogeneous study groups. Thus, no causal relationships between slacklining interventions and their effects could be derived. Hence, it was required to organize a well-designed longitudinal study for this purpose. Such a study needs to address the remaining questions pertaining to the specific influence of slackline training on behavioral improvements in balancing and spatial orientation. It is reasonable to expect that behavioral improvements in vestibular dependent path-integration and balancing [9] are coupled with associated neuroanatomical alterations. It remains unclear whether these effects would persist despite subsequent several months without training. Some previous studies have demonstrated retention of task-specific skills in response to training, [1,6,10,11], including balance training, but none of them reported a retention of the skill transfer, particularly in path-integration and general balancing ability.

Intact balance control is required not only to maintain postural stability but also to assure safe mobility-related activities during daily life [12,13]. Successful balancing and spatial orientation abilities require complex and harmonic processing of many inputs simultaneously, only one of which is the vestibular system. Neural pathways of these two abilities communicate through large networks and units of both cortical and subcortical structures [14,15]. In addition, falls represent a major burden to the society and earlier prevention treatments (in younger age) concerning this problem are also strongly encouraged [16].

The assumption of a vestibulo-hippocampal dependency is supported not only by previous research on relationship between the vestibular and the medial temporal lobe orientation systems, which revealed multiple pathways that exist between the two [17], but also by a disruption in function of the latter when the input from the former ceases [18,19]. Previous neuroanatomical studies in humans showed that complete abolishment of vestibular input, due to acquired chronic bilateral vestibular loss, leads to atrophy in distinct medial temporal lobe areas [20]. Additionally, professionals who intensively make use of their vestibular system during their daily artistic performances, such as ballet/ice dancers and slackliners, have differently structured temporal brain regions, including the hippocampus, compared to nonprofessionals [21]. 

Thus, we attempted to answer here whether one month of intensive balancing (through slacklining) will cause sustained neuroanatomical (gray matter) effects in any of the associated regions and functional effects on path-integration and balancing. We hypothesized improvements and retention of balancing and spatial orientation abilities coupled with related neuroanatomical changes. We are not aware of any longitudinal balance training study so far that investigated all these effects in such a comprehensive longitudinal manner.

## 2. Experimental Section

### 2.1. Ethical Approval

This study was carried out in accordance with the recommendations of and was approved by the Ethics Committee of the Medical Faculty at the Otto von Guericke University (approval number: 156/14). Each participant signed a document of informed consent before the beginning of the study.

### 2.2. Subjects

The study sample was already described in detail in our previously published paper, where we reported the immediate postintervention behavioral data only [9]. In brief, fifty healthy young subjects were recruited for this study and randomly assigned (without stratification) into two groups, control (12 females and 13 males; mean age = 23.2 years; SD = 2.6 years) and training (11 females and 14 males; mean age = 24.4 years; SD = 2.7 years). The two groups did not significantly differ in any of the recorded demographic and other characteristics, including age, height, weight, years of education and handedness. Physical activity was assessed by asking subjects how many hours they spent on sports weekly on average in the past three years; all sports were taken into consideration, including jogging, various team sports and cycling, excluding walking. Participants of both groups were paid the same amount of money for their participation in the study. 

Eligible subjects for this study were all those aged from 18 to 30 years who had no previous experience in slacklining or similar activity (i.e., highly demanding balancing activities, such as ballet dancing and rhythmic gymnastics) and had normal or corrected to normal vision. Exclusion criteria were injuries to the musculoskeletal system and systemic diseases (e.g., cardiovascular, metabolic and nervous system diseases). 

### 2.3. Study Design

**Figure 1:**
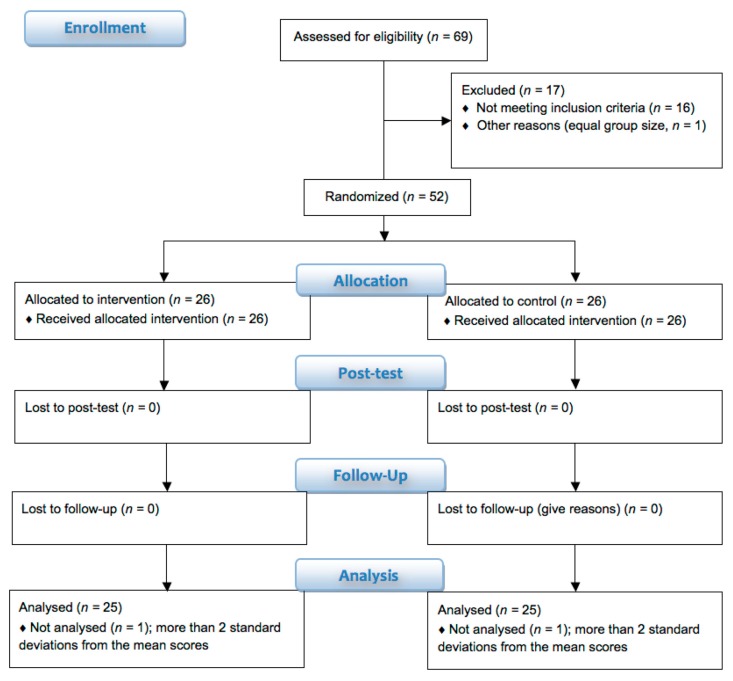
Consort flow-diagram of the study.

A flow diagram of the study is shown in Figure 1. This study was planned and organized as a randomized controlled single-blinded trial with factorial design (factors: time and group). Participants were randomly assigned to the training and control groups using a computer-based randomization procedure (Research Randomizer. Available online: https://www.randomizer.org). The computer-based randomization and assignment of participants to groups were performed by M.D. (not involved in data collection), with all other investigators blinded to the outcome of the randomization. Collection of data was performed by P.M., who was blinded to the subsequent statistical analysis.

The study involved measurements at three timepoints: baseline (pretraining), one month (±2 days) after baseline (i.e., directly after training, namely post-training) and three months (±2 days) after baseline (i.e., two months after training had been accomplished, namely follow-up). All trainings took place in the movement lab of our institute (German Center for Neurodegenerative Diseases) from February to April 2015. The rationale for the timing of assessments was based on the study by Taubert and colleagues from 2010 [11], since we also wanted to investigate the short- and medium-term effects of our intervention. 

### 2.4. Intervention

The detailed intervention procedure has already been described in our previously published work [9]. Briefly, during a one-month period the training group underwent intensive balance training consisting of 12 training units (three trainings per week with each training lasting 1 h; max. two consecutive nontraining days) on a 3-m-long slackline (“Power-wave 2.0” slackline rack), whilst the control group was instructed to abstain from any type of similar activity; the abstinence from this type of activity was confirmed by control group participants at the post-test. The training group had no previous experience with this or any activities of a similar type (balancing) and started the training from a very beginner level—to confirm this, on the first training day the participants were tested if they could stand or walk independently on the slackline. 

The trainings were led and supervised by an experienced instructor, whose assignment was to achieve the best possible skill level in the training group participants and to demonstrate the correct way of performing this skill. Each training unit consisted of a 10-min warm up session and a 50-min training session. The maximum group size allowed was four participants, so the instructor could dedicate enough time to each trainee. Moreover, the trainings were highly individualized, according to the skill and progression levels of each of the participants. The three training days per week (Monday to Friday) were scheduled according to the availability of participants, with the condition of having trainings on a maximum of two consecutive days (there must have been at least one day break before the third training). Our independent coach provided subjects with verbal feedback of task achievement. The aim of slackline training protocol was an individually optimal performance outcome at the end of the four-week period.

The slackline tension was individualized—in the middle portion it was adjusted in such a way that the distance to the metal bar underneath remained at least several centimeters when participants stood on it. The purpose of such adjustment was to keep the training at a higher level of difficulty by maintaining the slackline slack and unstable rather than tight and stable. Moreover, the slackline length was intentionally chosen to be 3 m, so that a higher rate of turns on the slackline could be achieved, corresponding to a higher rate of semicircular canal stimulations. 

### 2.5. Behavioral Tests

#### 2.5.1. Clinical Balance Test (CBT)

The CBT has been described in detail in our previously published work [9]. In brief, the CBT consisted of standing on stable and unstable surfaces and walking conditions, all of which further contained subconditions with open and closed eyes. All conditions in their exact order of administration are provided in Table 1.

The maximum amount of points that could be collected on the test was 90, with each condition carrying the minimum of 0 and the maximum of 3 points. In each of the standing conditions participants were instructed to maintain the required position for 15 s, whereas in walking conditions there was no time requirement and participants were asked to walk at their own pace. Participants’ stability was assessed as follows:

3 points—stable stance, absence of body movements;
2 points—stable stance, attempted to stabilize body using arm movements;
1 point—stable stance, attempted to stabilize body using arm and leg movements;
0 points—loss of stance.

The assessment was based on the subjective opinion of trained assessors; the inter-rater reliability of the test (determined with ICC coefficient) was 0.98 ± 0.04 (SEM = 0.003).

#### 2.5.2. Triangle Completion Test (TCT)

For assessment of nonvisual spatial orientation, the triangle completion test (TCT) from our earlier work was used [9,22]. Briefly, six triangular paths in total were marked on the floor, three in each direction (left and right), with turning angles of 60°, 90° and 120°. Both active-walking and passive-wheelchair conditions were applied in the test. In the active-walking condition participants were guided on foot while holding onto a wooden bar. In the passive-wheelchair condition were seated and pushed in a standard wheelchair. There was only one repetition per participant for each condition and path, thus 12 trials per participant were recorded in total (3 to the left and 3 to the right, times 2 conditions). In each trial, after the participant had walked or had been pushed in the wheelchair along two sides of triangle, he or she had to return back to the starting point, using the shortest possible route (hypotenuse). The main outcome variables were: (a) the distance error on each trial (distance from the participant’s stopping point to the starting point); (b) the angular error (angular deviation from the optimal direction towards the starting point). Participants were blindfolded in a quiet room during the test, so they were not receiving any visual or auditory cues. It can thus be assumed that the only cues they could use were somatosensory and vestibular in the active-walking condition and vestibular only in the passive-wheelchair condition.

### 2.6. MRI

MR images were acquired on a 3 Tesla Siemens MAGNETOM Verio scanner (Syngo MR B17) using a 32-channel head coil. High-resolution T1-weighted MPRAGE sequences were acquired using a 3D magnetization-prepared rapid gradient echo imaging protocol (224 sagittal slices, voxel size: 0.8 × 0.8 × 0.8 mm^3^, TR: 2500 ms, TE: 3.47 ms, TI: 1100 ms, flip angle: 7°).

Voxel-based morphometry (VBM) is a whole-brain unbiased technique for analysis of regional gray matter volume and tissue changes (Ashburner and Friston, 2000). Preprocessing and analysis were done using the VBM8-toolbox and it involved gray matter segmentation, template creation via DARTEL, spatial normalization to standardized Montreal Neurological Institute (MNI) space and smoothing with a Gaussian kernel of 8 mm full width at half maximum (FWHM). Modulation step was applied, so the obtained results represent relative differences in regional gray matter volume (GM), corrected for individual brain size. Total intracranial volume (TIV) was included as a covariate in the analysis. 

### 2.7. Outcome Variables and Data Analysis

In order to analyze the difference in gray matter volume changes between groups, a 2 × 3 full-factorial design with the factors group (training, control) and time (pre-training-test, post-training-test and follow-up) was applied. The subsequent within-training-group analysis involved a paired *t*-test procedure. Multiple comparison correction was applied in forms of false-discovery-rate (FDR) at voxel and cluster levels—results were considered significant at *p* < 0.05, unless otherwise specified. The variance in results is reported as 95% confidence interval (2 standard errors of the mean) of the difference. The outcome variable for the neuroanatomical analysis was the structural change in brain neuroanatomy as observed by VBM. Data were analyzed with MATLAB (Mathworks, Natick, MA, USA) and SPM12 (UCL, Great Britain, UK). Analysis of the behavioral data was performed with SPSS v.21 (IBM, Armonk, NY, USA). The results of the VBM analyses were visualized using the xjView toolbox (http://www.alivelearn.net/xjview). VBM data of 11/25 participants form the eyes-open (EO) group in this study have already been published in Dordevic et al., 2018, where they were compared to the eyes-closed (EC) group. For display purposes, statistical maps are overlaid on the Colin Holmes 27 (ch2) template of the international consortium for brain mapping (ICM); figures are presented in neurological convention: R = R, L = L.

## 3. Results

The final analysis included 25 participants in each group. Two participants (one from each group) were not considered for the analysis because of major outliers, reaching more than 2 standard deviations above the mean score of all participants at the orientation test, whereby their error was larger compared to the group mean. All subjects were recruited from December 2014 until March 2015 and their characteristics are shown in Table 2. 

### 3.1. Training-Specific Skills

**Figure 2:**
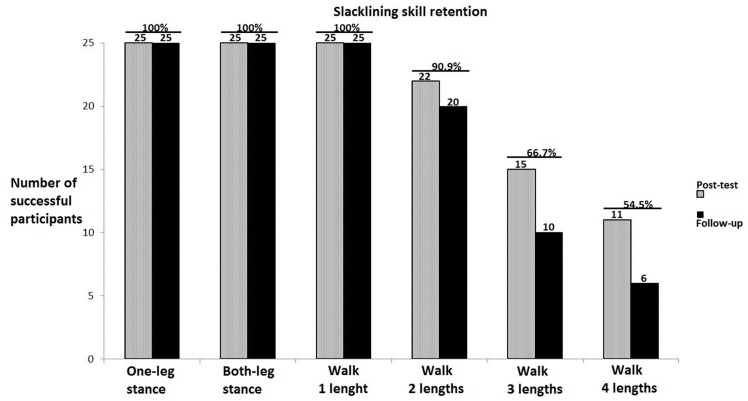
Task-specific skill level at the post-test and follow up.

Figure 2 depicts the task-specific skill level participants achieved at the post-training-test (gray columns) and the follow-up test (black columns). While at the pre-training-test none of the participants was able to stand on the slackline for 5 s, at the post-training-test all of them could stand on one and both legs (for the minimum of 5 s), turn and walk on the slackline Most of the participants could walk at least two lengths (6 m with turn) by the end of the training period. The figure illustrates a gradual decrease in retention of task-specific skills with the task difficulty; that is, more difficult the task, lower the number of participants who could perform at the post-training-test level, when tested at the follow up. 

### 3.2. Triangle Completion Test (TCT)

**Figure 3:**
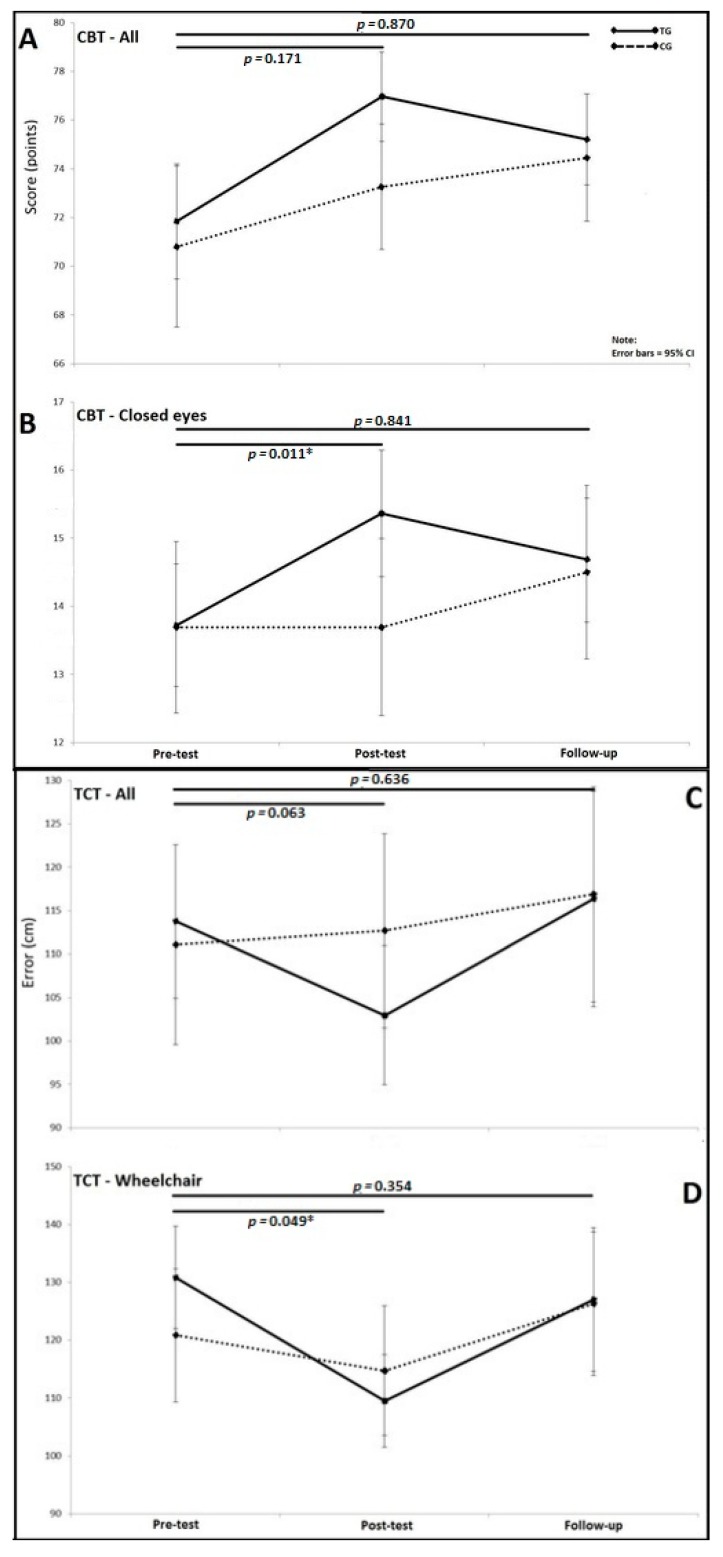
Score on clinical balance test (CBT) and error on the triangle completion test (TCT) for both the training and the control group from post-test and two-month follow-up. A—score on all conditions of the CBT; B—score on closed eyes conditions only of the CBT: C—error in distance on all conditions of the TCT; D—error in distance on wheelchair conditions only of the TCT.

Post-test results of the clinical balance test (CBT) and the triangle completion test (TCT) from pre-training-test to the post-training-test have already been reported in our previous publication [9]. The novel results pertain to the third timepoint (follow-up), which are shown in the Figure 3. As illustrated, none of the significant interaction effects that were observed from pre- to post-test on the CBT and TCT were present at the two-month follow-up. This was mainly caused by worsening of the training-group participants’ performance from the post-training-test to follow-up test. At the follow-up, the training group had slightly better score then the control group for CBT for all conditions (75.2 ± 4.2 vs. 74.4 ± 5.1, F(1,48) = 0.013, pη^2^ = 0.0001) and CBT for closed-eyes conditions (14.7 ± 2.1 vs. 14.4 ± 2.4, F(1,48) = 0.303, pη^2^ = 0.006) (Figure 3). For the TCT, the difference virtually did not exist between the training and control groups, both when all conditions were analyzed (116.4 ± 74.3 vs. 116.9 ± 78.6, F(1,47) = 0.126, pη^2^ = 0.003) and also when only wheelchair condition was analyzed (127.0 ± 72.7 vs. 126.3 ± 81.1, F(1,47) = 0.680, pη^2^ = 0.014). 

### 3.3. Voxel-Based Morphometry (VBM)

#### 3.3.1. Interaction Effects

**Figure 4:**
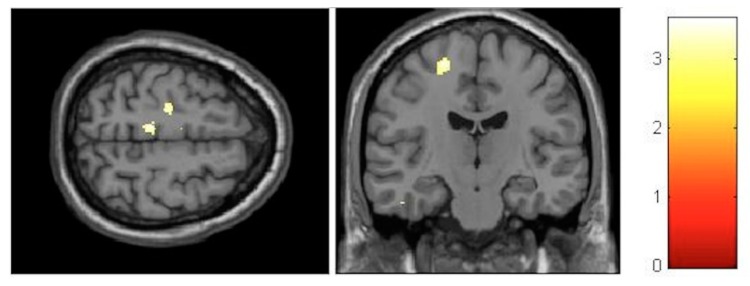
Voxel-based morphometry (VBM) observed gray matter volume (GM) increases for the group–time interaction from pre- to post-test.

**Figure 5:**
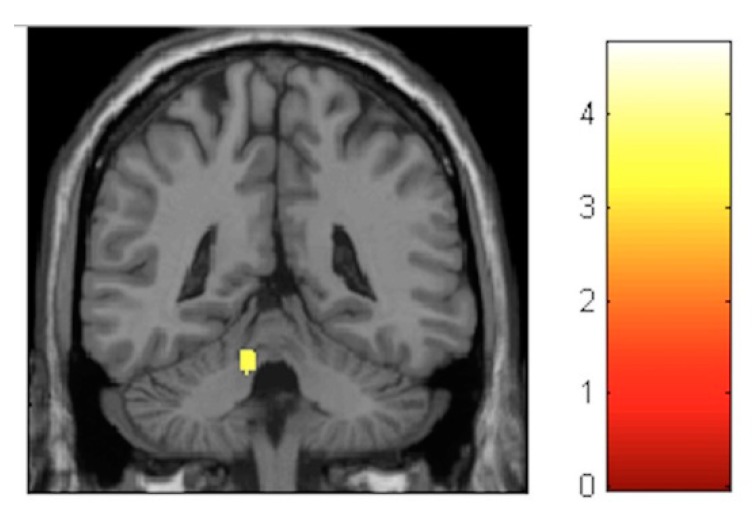
VBM-observed GM decreases for the group–time interaction from pre- to post-test.

For the VBM analysis, the interaction effects were firstly investigated and revealed no significant results at the FDR-corrected level. At the post-test, the largest increments were observed in the left paracentral lobule (T = 3.56, *p* < 0.001, FDR = 0.782, effect size (D) = 0.35) (Figure 4), whereas, the largest decrements could be seen in the left cerebellum (T = 3.91, *p* < 0.001, FDR = 0.672, effect size (D) = 0.36) (Figure 5). 

**Figure 6:**
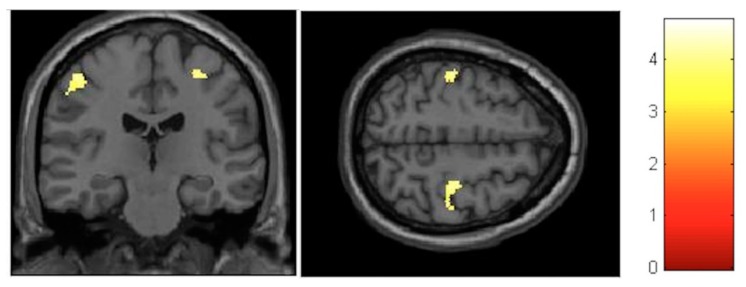
VBM-observed GM increases for the group–time interaction from pretest to follow-up.

**Figure 7:**
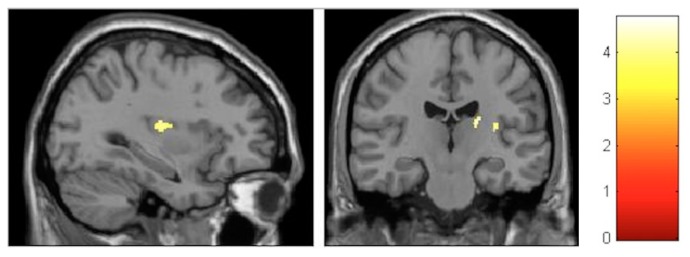
VBM-observed GM decreases for the group–time interaction from pretest to follow-up.

Although not significant at the FDR-corrected level, the interaction effects were stronger at the follow-up compared to the post-test. The largest increase in gray matter was observed in the paracentral lobules bilaterally (T = 4.04, *p* < 0.001, FDR = 0.217, effect size (D) = 0.37) (Figure 6). The largest decrease was found in the right posterior insula and right thalamus (T = 4.33, *p* < 0.001, FDR = 0.171, effect size (D) = 0.38) (Figure 7). 

Despite the fact that no significant p-values after correction could be found, the effect sizes indicated small effects, therefore prompting further consideration in within-group analyses.

#### 3.3.2. Within-Training-Group Effects

**Figure 8:**
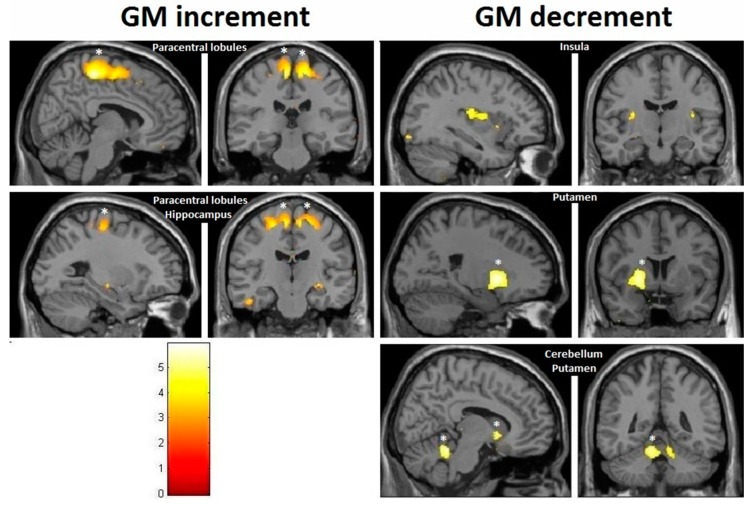
VBM-observed GM increases and decreases in the training group from pre- to post-test; * False-discovery-rate (FDR)-corrected.

**Figure 9:**
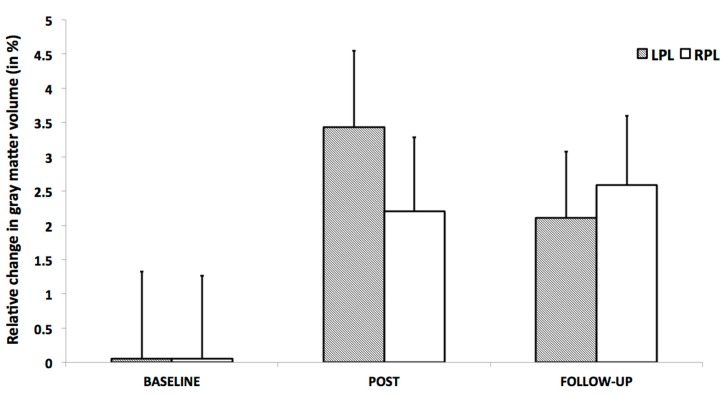
Relative gray matter (GM) change in the peak voxel in the left (LPL) and right (RPL) paracentral lobules for all participants of the training group over the three timepoints. The column-chart shows the mean change in GM from baseline for each timepoint and side. Baseline columns represent zero level. Error bars represent 2 standard errors of mean (SEM).

Considering our hypotheses and observed effect sizes from the interaction analyses, we proceeded with within-training-group analyses and therefore performed paired *t*-tests over time. We found a very strong effect in the training group even after FDR correction. Moreover, paracentral effects were localized in virtually the same brain regions as those observed in the group–time interaction analysis (with the effects being significant after FDR correction) (Figure 8). As illustrated, at the FDR-corrected voxel-level (marked with asterisk), gray matter volume (GM) increase was significant in the sensory-motor cortex bilaterally, whereas a significant decrease was found in the left putamen and cerebellum (Figure 4). Additionally, at the uncorrected (*p* < 0.001) level, GM decrease was found in the bilateral cerebellum, the bilateral insula and the bilateral putamen, and increase could be seen in the bilateral paracentral lobules, the right hippocampus and the left inferior temporal lobe (Figure 9). Respective MNI coordinates and cluster sizes are given in the Table 3. Moreover, a relative change in gray matter volume of the paracentral lobule bilaterally is depicted in Figure 9. As shown, from pre- to post-test there was a 3.43 ± 1.12 percent change on the right side and 2.10 ± 0.97 percent change on the left side.

**Figure 10:**
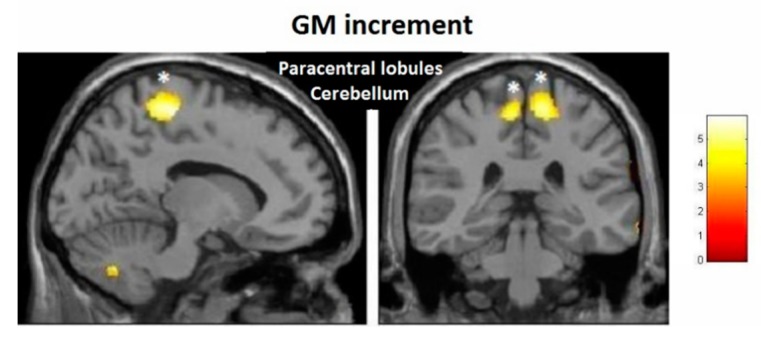
VBM-observed GM increases in the training group from pretest to follow-up; * FDR-corrected.

**Figure 11:**
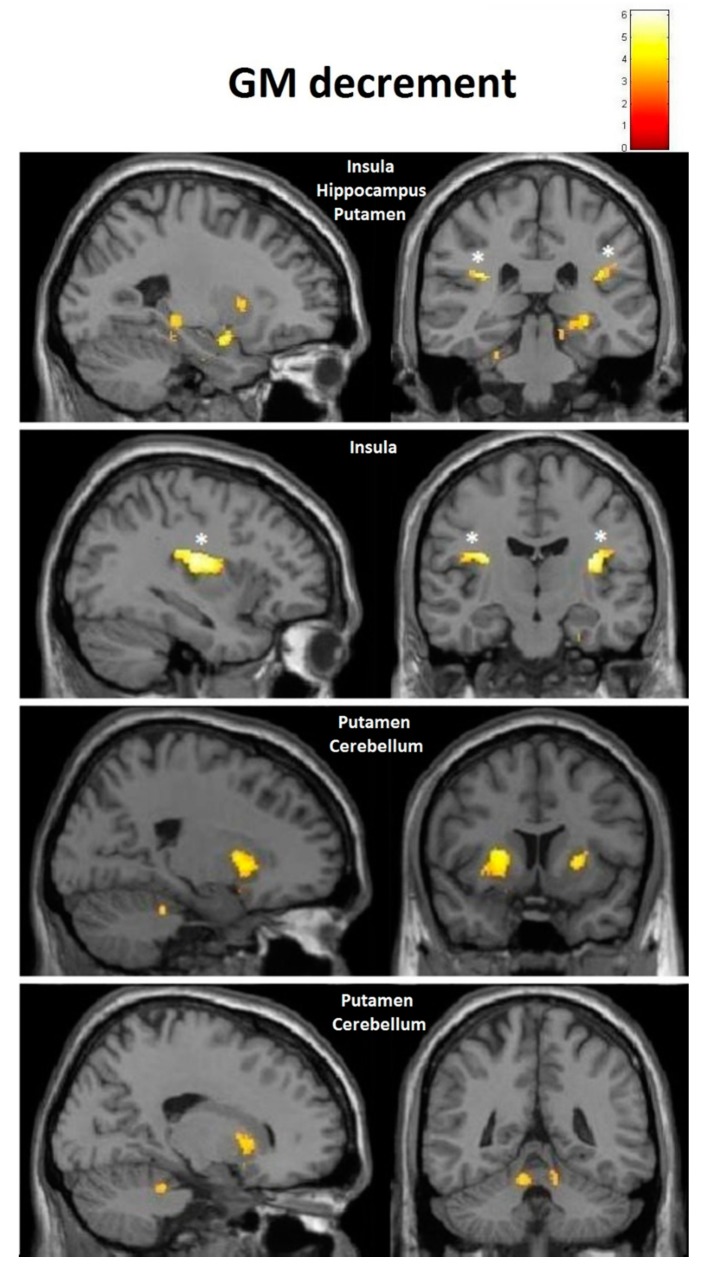
VBM-observed GM decrease in the training group from pretest to follow-up; * FDR-corrected.

At the follow-up, within-training-group analysis revealed significant gray matter changes at the FDR-corrected voxel-level in both directions (Figure 10 and Figure 11, asterisk), with increases in GM within the bilateral paracentral lobules (Figure 10) and decrements in the insula bilaterally (Figure 11). Furthermore, at the uncorrected level we observed decrements in the cerebellum and the putamen (Figure 11). In contrast to the small increments displayed at the post-test, the right hippocampus manifested decrements at multiple sites at the follow-up. Respective MNI coordinates and cluster sizes are shown in Table 4. A relative change in gray matter volume of the paracentral lobule from baseline to follow-up was 2.20 ± 1.08 percent change on the right side and 2.59 ± 1.01 percent change on the left side (Figure 9).

## 4. Discussion

The current results do not confirm our hypothesis on a sustained far transfer of slackline training on balancing and path integration abilities. That is, although one month of intensive slackline training led to improvements in balancing and path-integration abilities immediately after the training, these were entirely lost at the follow-up testing. With regards to the neuroanatomical effects of the slackline training, we could not find any significant interaction effects immediately after the training or at the follow-up. The only significant neuroanatomical changes could be observed within the training group, which prompts their cautious interpretation. 

Improvements in vestibular-dependent spatial orientation and balancing skills immediately after the training indicate that these effects can potentially be attributed to enhancement of the vestibular system’s function in response to the training itself [9]. According to a meta-analysis of balance trainings, it is sufficient to perform three trainings over four weeks, with each training lasting at least 10 min, to expect significant improvements in balance [23]. Another meta-analysis on slackline trainings in particular revealed high task-specific effects and low to moderate non-task-specific effects [11]. This is in accordance with our earlier findings at the post-test [9], where our participants had learned how to slackline and achieved small to medium effect size for the improvements on the clinical balance test. According to another recent meta-analysis on dose-response effects of balance trainings, one has to undergo about three months of balance training with frequency of three to six trainings per week for the optimal training effects [24]. We can confirm from our data that room for further improvement still existed after our one-month intervention, even in the task-specific skills. Considering that our participants were involved in an intervention lasting less than three months, this could have possibly led to their inability to retain the transfer effects for another two months of not practicing. Erickson et al. further suggested that exercise interventions should last at least 6 to 12 months for pliable effects related to the hippocampus [25]. 

The main focus of this paper was to describe the neuroanatomical changes, which have been shown previously to occur very fast in response to balance training [2] and also to exert a temporal dynamic pattern in various cortical regions [1]. In our study, however, these were not significant at the interaction level, both at the post-test and follow-up. Therefore, we can only discuss the findings of the within-training-group analysis. The within-group changes did match well with previously described regions that are active in response to stimulation of the vestibular system [26]. The observed increments in gray matter volume (GM) for bilateral sensory-motor cortex could be anticipated, given the role of this region. GM decrements took place in many regions known to be receiving vestibular input and/or to be responsible for movement control, such as the cerebellum, the insula and the putamen. Decrements in various brain regions, including the putamen, were also reported by earlier studies [27,28]. Such an outcome could perhaps be well explained as an effect of multisensory interaction that also takes place during slackline training. The finding that regions responsible for somatosensation and movement initiation [29,30] “enlarged” while those for vestibular sensation (insular region [14,31]) “shrunk” could be due to the fact that the somatosensory function becomes significantly enhanced when the vestibular system is active, as demonstrated by several studies by Ferrè et al. [32,33,34]. When both systems are active simultaneously, instead of having a supra-additive effect, the vestibular system actually becomes a modulator of a gain in somatosensation [34]. The training effect on this complex interaction resulted in an improvement in both somatosensory and vestibular functions, which was lost two months later, when the most significant decrement of GM volume in the insula was observed bilaterally. Interestingly enough, even conditions such as vestibular neuritis lead to an increase in GM volume in insular vestibular cortex [35,36], revealing possible vestibular compensation mechanisms in response to a loss of sufficient vestibular input. Cortical structures receiving vestibular and somatosensory inputs are well interconnected [5,37,38,39], with both excitatory and inhibitory influences being exerted among themselves. These could have also contributed to the effects we observed. Besides that, somatosensory cortices have also been linked to processing of higher-order vestibular sensations [40], not only of somatosensory ones. In addition, recent results suggest that the core of the vestibular cortex, the parieto-insular vestibular cortex (PIVC), consists of a posterior visual–vestibular part and an anterior vestibular-only part [41,42,43]. The posterior visual–vestibular part appears to be particularly important for visual–vestibular integration [44,45,46]. It could be that the decrements we observed in this region specifically target the visual–vestibular part, since learning to slackline involves the integration of both visual and vestibular cues.

Subcortical areas involved in movement control and planning, including the anterior cerebellum and the putamen, demonstrated a persistent GM reduction in our study. Cerebellar volume reductions in ballet dancers were reported by Nigmatullina and colleagues [47] and were also confirmed by our own study on ballet dancers [22]. The anterior cerebellum is known to contain a homunculus representation and receives vestibular input [48,49], while the putamen receives inputs from all cortical and subcortical structures involved in motion and is also activated by vestibular system stimulation [26,50]. In contrast to previous belief, it is now known that direct connections between the cerebellum and the basal ganglia exist, with the cerebellum providing supervised learning and the basal ganglia (mainly the striatum) concerned with reinforcement of learning [39]. Of the striatal region, in contrast to the caudate, the putamen is primarily linked to primary and associative somatosensory and motor regions, making it crucial for motoric activities [51]. Since subcortical structures are known to be the main sites where well-learned movement patterns and sequences are stored and controlled, it could be that one month of training did not suffice for this new skill to become automatized, leading to consistent activation of the cortical sensory-motor regions and their subsequent volume enlargement. 

The vestibular system projects to the hippocampus, activating it functionally, and it is shown to have an important function for spatial orientation and learning [14,17,52,53]. Humans can store spatiotemporal dynamic patterns of motion and retrieve them completely using vestibular and somatosensory cues [52,54]. However, we found no significant effects in the hippocampus in our study. Those nonsignificant effects were present in the right hippocampus, in accordance with several studies showing that spatial orientation is mainly processed by the right hippocampus [55,56]. 

We could clearly observe a dissociation between the structural (neuroanatomical) effects and functional far-transfer effects in the training group. While the neuroanatomical effects persisted even two months after the last training session, functional far-transfer gains were completely lost. However, this was not the case with specific slacklining abilities. One possible empirical way of explaining this would be that traces of a far-transfer skill, in this case path-integration (TCT) and balancing (GGT), remain intact in the brain for longer periods of time to facilitate the relearning process at some later point. Earlier, an interdependency between the efficiency, durability and transferability of training has been suggested [57]. The observed loss in far-transfer behavioral skills (TCT, GGT) in our study could be due to an insufficient duration of training, since the specific skills (slacklining abilities) remained at a much higher level compared to the far-transfer ones. 

Vision is known to be an important factor in multisensory interaction. For instance, it has been shown that during optokinetic stimulation and central fixation, which were very common in our intervention, visual cortices became active, whereas the activation of vestibular ones (bilateral insula) was being suppressed [58,59,60,61]. Indeed, the concept of vestibular compensation is well known to rehabilitation specialists, whereby the adaptation of the vestibular system to inputs from visual and somatosensory systems is used for the purpose of improving patients’ recovery [62]. Thus, the learning during training itself was highly dependent on vision, as the participants had their eyes open, and this could have in turn influenced all our expected results on changes within the vestibular-related brain regions, since visual information is a crucial component of spatial representation [63]. All the inputs related to spatial navigation might have been integrated in the thalamus [64] during training and then, as such, affected the subsequent outcome on behavioral tests. Such bias can easily be removed by running a similar study where participants’ visual input would be blocked, which we will take into consideration for our future work. Another major limitation of our study lies in the protocol which included only one post-test and one follow-up; this did not allow us to track the temporal dynamics of training effects, which have previously been shown to exist [11]. However, the neuroanatomical changes where quite stable over time, with almost the same regions showing significance at both timepoints. Another limitation is the relatively small sample size, one of the main factors not allowing for more significant interaction effects at the corrected level. Interaction effects after FDR correction also could not be found by other studies that attempted to answer similar questions and applied similar methodology [1]. It has already been shown that results of voxel-based morphometry rely highly on various factors, such as preprocessing steps and the software package used, thereby producing heterogeneous outcomes and diverse significances [65]. Nevertheless, observing significance levels only may be misleading [66,67], and our effect sizes revealed small effects despite insignificant interaction effects. Information about participants’ physical activity level was collected, but they were not asked about the amount of their walking activity, which should also be corrected by future studies. In addition, having only a passive control group could not exclude the possibility that motivational and social factors have contributed to the observed effects. More research in this direction is required to confirm our findings and contribute to better understanding of the effects of similar interventions.

## 5. Conclusions

In conclusion, the immediate effects of one-month slackline training on path integration and balancing skills were entirely lost at the follow-up. We could not find any significant neuroanatomical interaction effects; these were constrained rather to within-training-group effects. Changes in gray matter volumes within the training group corresponded well to the regions known to receive vestibular inputs and included increments in sensory-motor paracentral lobules and decrements in insular cortices, putamen and cerebellum, with the hippocampus showing nonsignificant changes. The obtained combination of brain changes can be perhaps explained by a complex interaction of vestibular, somatosensory and visual systems that occurs during training, together with insufficient training time for the skill to become automatized, thus emphasizing the functions of sensory-motor cortical areas. While the neuroanatomical changes in the training group were retained even after two months of not practicing, the behavioral transfer effects were entirely lost, indicating that although the traces of newly learned skill remain in the brain, the drop in performance of related tasks can be observed. For retention of behavioral skills, a longer training period might also be necessary. 

## Figures and Tables

**Table 1:** Conditions of the clinical balance test (CBT) ([9]).

No.	Condition	Task		Points (Min = 0, Max = 3)			
				0	1	2	3
1.	Static—stable surface (floor)	Stand with feet together—open eyes					
2.		Stand with feet together—closed eyes					
3.		One leg stance—left—open eyes					
4.		One leg stance—right—open eyes					
5.		One leg stance—left—closed eyes					
6.		One leg stance—right—closed eyes					
7.	Static—unstable surface (pad)	Stand normally (hip width stance)—open eyes					
8.		Stand with feet together—open eyes					
9.		Stand normally (hip width stance)—closed eyes					
10.		Stand with feet together—closed eyes					
11.		One leg stance—left—open eyes					
12.		One leg stance—right—open eyes					
13.		One leg stance—left—closed eyes					
14.		One leg stance—right—closed eyes					
15.	Dynamic	Walk inside the zone (4 m × 30 cm)	Forward				
16.			Turn (90°)				
17.			Backward				
18.		Walk on the line (4 m × 5 cm)	Forward				
19.			Turn (90°)				
20.			Backward				
21.		Walk on the line with feet one after the other (4 m × 5 cm)	Forward				
22.			Turn (90°)				
23.			Backward				
24.		Walk on the beam (4 m × 10 cm)	Forward				
25.			Turn (90°)				
26.			Backward				
27.		Walk on the beam sideways (4 m × 10 cm)	Rightward				
28.			Turn (90°)				
29.			Leftward				
30.		Walk on the line with closed eyes (4 m × 5 cm)	Forward				

**Table 2:** Characteristics of participants.

Characteristic	Training (*n* = 25)	Control (*n* = 25)
Age (years)	24.5 ± 2.7	23.2 ± 2.6
Sex (females)	11 (44%)	12 (48%)
Weight (kg)	69.1 ± 12.5	65.0 ± 10.0
Height (cm)	173.4 ± 9.2	170.3 ± 8.4
Hours of activity (per week)	3.0 ± 1.8	3.2 ± 2.5
Handedness (right)	24 (96%)	23 (92%)
Profession (student)	22 (88%)	23 (92%)
Suffered a small injury (e.g., ankle sprain)	5 (20%)	5 (20%)
Ethnic origin		
European	20 (80%)	19 (76%)
Asian (Indian)	5 (20%)	5 (20%)
Arabic	0 (0%)	1 (4%)

**Table 3:** Montreal Neurological Institute (MNI) coordinates (x, y, z) of VBM-detected gray matter changes in the training group from pretest to post-test.

Effect	Brain Region	Left Hemisphere MNI Coordinates (Cluster Size in Voxels)	T	d	Right Hemisphere MNI Coordinates (Cluster Size in Voxels)	T	d
Increment	Paracentral	−9, −32, 59 (1298)	5.93	0.41	14, −36, 63 (1589)	5.47	0.40
Increment	Hippocampus				34, −16, −11.5 (20)	3.85	0.36
Decrement	Insula	−32, −10, 12 (60)	3.91	0.36	35, −20, 18 (261)	4.36	0.38
Decrement	Putamen	−20, 14, 0 (1879)	5.45	0.40	24, 11, 2 (137)	4.13	0.37
Decrement	Cerebellum	−12, −47, −21 (511)	5.33	0.40	11, −47, −23 (262)	4.06	0.37

**Table 4:** MNI coordinates (x, y, z) of VBM-detected gray matter changes in the training group from pretest to follow-up.

Effect	Brain Region	Left Hemisphere MNI Coordinates (Cluster Size in Voxels)	T	d	Right Hemisphere MNI Coordinates (Cluster Size in Voxels)	T	d
Increment	Sensory-motor	−8, −32, 60 (314)	5.17	0.39	11, −29, 60 (1087)	6.09	0.41
Decrement	Hippocampus				27, −30, −6 (133) 23, −2, −17 (119)	4.06 4.74	0.37 0.38
Decrement	Insula	−33, −12, 18 (586)	5.57	0.40	33, −17, 17 (797)	6.17	0.41
Decrement	Putamen	−26, 8, 6 (631)	4.72	0.38	21, 11, 2 (134)	4.22	0.38
Decrement	Cerebellum	−11, −45, −23 (245)	3.84	0.36	11, −65, −23 (224)	3.99	0.37

## Data Availability

Data used in this study belong to German Center for Neurodegenerative Diseases (DZNE). For external institutions a special permit is required for obtaining these.
